# Experiences with facility delivery services within the context of a maternal neonatal health project in Gombe State, Northeast Nigeria: a qualitative study

**DOI:** 10.11604/pamj.2025.51.16.43132

**Published:** 2025-05-14

**Authors:** Maryam Al-Mujtaba, Olukolade Shobo, Bolanle Christy Oyebola, Benson Ohemu, Isaac Omale, Abdulrahman Shuaibu, Jennifer Anyanti

**Affiliations:** 1 Duke University School of Nursing, Duke University, Durham, North Carolina, United States of America,; 2 Monitoring and Evaluation Department, Society for Family Health, Abuja, Federal Capital Territory, Nigeria,; 3 Programmes Department, Society for Family Health, Abuja, Federal Capital Territory, Nigeria,; 4 Communications Department, Society for Family Health, Abuja, Federal Capital Territory, Nigeria,; 5 Office of the Executive Secretary, Gombe State Primary Healthcare Development Agency, Gombe, Gombe State, Nigeria,; 6 Office of the Deputy Managing Director, Society for Family Health, Abuja, Federal Capital Territory Nigeria

**Keywords:** Facility delivery, maternal healthcare, maternal health project, Northeast Nigeria

## Abstract

**Introduction:**

a maternal neonatal health (MNH) project implemented in Gombe State improved uptake of facility delivery services from 27% to 65%. The project supplied health commodities to health facilities, provided women with cost-free transportation, and implemented the Village Health Worker program. Village health workers are lay indigenous women trained to provide community-based maternal and newborn care and to facilitate linkage to health facilities. We explored women's experiences with facility delivery services within the context of the MNH project.

**Methods:**

qualitative data were obtained through focus group discussions with women who delivered within the last 12 months. Participants were asked questions related to their experiences with the access and use of facility delivery services. Data were organized with NVivo 12 (Pro for Windows) and analyzed using directed content analysis.

**Results:**

six focus group discussions were conducted with 58 participants. Mean age was 25.1 (± 5.3) years old. All the women preferred facility delivery over home delivery for quality care. Most women reported experiencing immediate and respectful care with facility delivery services, and healthcare workers' competence and attitude were more important than the gender of the healthcare worker. However, for some women use of facility delivery services was limited due to the use of traditional birth attendants, absent husbands at the onset of labor, imminent delivery, long distance to the facility, expensive transportation fees, healthcare worker absenteeism and long clinic wait times.

**Conclusion:**

maternal neonatal health projects should be designed to ameliorate the effects of socio-economic and facility level factors that limit use of facility delivery services.

## Introduction

Sub-Saharan Africa is responsible for about two-thirds (66%) of global maternal mortality ratio (MMR) [1,2], and 41% of global neonatal mortality rate (NMR) [3]. Within sub-Saharan Africa, Nigeria has the highest MMR (917 deaths per 100,000 live births) [2] and the highest NMR (39 deaths per 1,000 live births) [4]. Within Nigeria, Gombe State, in the northeastern region of the country, has one of the highest MMR (1002 per 100,000 live births) and NMR (20.7 per 1000 live births) [5]. To achieve Sustainable Development Goal 3: of reducing global MMR to less than 70 per 100,000 live births, and reducing global NMR to as low as 12 per 1,000 live births [6], addressing the very high MMR and NMR in Gombe State in Nigeria is particularly vital.

The evidence-based strategy for reducing MMR and NMR is for women to give birth in a health facility and not at home [7,8]. This is because health facilities are expected to be staffed with skilled birth attendants (e.g. doctors, nurses, or midwives) who are trained to provide professional care to women and newborns during childbirth [9,10]. Unfortunately, in Nigeria, over 60% of women deliver at home [11]. Data from 2018 indicated that in Gombe State, only 21.2% of women delivered with the assistance of a skilled birth attendant. Those data indicated that 10.5% of women delivered with the assistance of non-skilled birth attendants (e.g. community health extension workers and traditional birth attendants), while 25.1% delivered their babies with no one present [4].

The main factors that limited use of facility-based maternal neonatal health (MNH) services in Gombe State, like in most sub-Saharan African countries, include socio-economic factors: financial and geographical inaccessibility, lack of support from significant others (mothers-in-law and husbands), and lack of access to appropriate information [12]. In Gombe, factors that strongly discouraged women from using facility delivery services were an unhygienic birth environment, lack of privacy during the birthing process, and unclear user fees [13]. In addition, the most important factor women considered when deciding on the place of delivery was the quality of the healthcare, followed by the absence of sexual, physical, and verbal abuse, respectively [13].

To improve the overall quality of maternal healthcare services and reduce the MMR and NMR in Gombe State, the Society for Family Health (a Nigerian non-governmental organization), in collaboration with the Gombe State Government through the State Primary Healthcare Development Agency (The Agency), implemented a maternal neonatal health (MNH) intervention project in the state from October 2016 to September 2018. The MNH project targeted the proximate causes (healthcare worker insufficiency, transportation barrier, and unavailability of essential lifesaving drugs) of maternal and neonatal mortality. The main components of the program included: 1) supply of essential commodities to facilitate improved quality of care; 2) village health workers (VHWs) - a cadre of selected indigenous women 15 years old or older trained to engage directly with families over health choices they make that affect maternal and neonatal survival and provide linkage to the facility; 3) charge-free emergency transport to health facilities for women in labor [14].

After two years of MNH program implementation, facility delivery services increased from 27% to 65% [15]. Considering the MNH program significantly improved the use of facility delivery services, we considered it vital to explore the experiences of women beneficiaries of the MNH program. We also believed that qualitative findings could help explain the MNH program´s quantitative outcome metrics and highlight which components/characteristics of the program were more appealing or not to the women. Therefore, this study aimed to explore the experiences of women with facility delivery services within the context of the MNH project in Gombe State.

## Methods

**Study design:** this is a qualitative descriptive study design [16], to evaluate the MNH project via data and rich descriptions of birth experiences directly from the women who have used the MNH program via focus group discussions.

**Study setting:** this study was conducted in Akko, Banganje North, and Zange wards of Gombe state from 30^th^ October to 1^st^ November 2018. The state is predominantly rural [17], has 12 ethnic groups, with the Hausa language as the inter-ethnic medium of communication [18]. Most residents (72.2%) live on under USD1/day [19], and literacy rates are 37.5% and 47.5% among females and males, respectively [17]. The state has 605 health facilities [20]. Four hundred and eighty-six (486) of the public health facilities provide labor and delivery services, out of which 460 are primary health facilities and 114 are referral facilities [21]. The state´s total healthcare worker density is 1 per 1,000 [19]. This density is lower than the national health worker density of 2.52 per 1,000 [22], and much lower than the WHO standard of 4.45 per 1,000 population [23].

**Study participants:** women beneficiaries of the MNH program in Gombe State who have delivered either at home or at the facility within the 12 months preceding the time of study, residing in one of the study-selected wards: Banganje North, Akko, and Zange.

**Variables:** home delivery, facility delivery, facility delivery wait time, healthcare workers´ gender and attitude, and traditional birth attendants.

**Sampling:** there is no formulae for calculating sample size for a qualitative study, however, 8-10 people per group is recommended [24]. For this study, using purposeful sampling [25], we recruited 10 participants per group. Ten participants per group is considered large enough to gain a variety of perspectives and small enough not to become disorderly or fragmented [26].

**Bias:** to minimize bias, we employed the maximum variation sampling, a technique that enables the selection of participants with varying levels of facility delivery service utilization [27]. This sampling approach ensured diversity by including groups across the spectrum of facility usage. We selected three geographical wards from the 57 wards where the MNH program operated, representing high (Banganje North, 96%), medium (Akko, 65%), and low (Zange, 23%) utilization rates of maternal healthcare services. We chose to focus on wards because they serve as the primary operational units for healthcare implementation, typically containing 10,000 to 30,000 people [28], maintaining political homogeneity, and having clearly defined boundaries [12]. Study eligibility was restricted to women who had given birth either at home or in a health facility within the previous 12 months.

We conducted two focus group discussions (FGDs) per ward. The first group (facility group) was to consist of women who had their last delivery at the health facility, and the second (home group) was to consist of women who had their last delivery at home. We were able to obtain eligible participants for both the facility group and the home group in two wards (Akko and Zange). However, we were unable to get a home group in the last ward (Banganje North), where facility delivery uptake was 96% (almost 100%). Participant recruitment was stopped once a target of 10 women was reached for each FGD. Participants who agreed to participate in the FGDs met with researchers at an appointed date and time. The first 4 FGDs had 10 participants per group, while the fifth and sixth groups had 7 and 11 participants, respectively. The fifth group had fewer than 10 participants (7 participants), because three participants had to attend to farm harvesting tasks. Conversely, the sixth group included more than 10 participants (11 participants) because one of the three participants who was unavailable for the fifth group was available to join the sixth group. In total, we conducted 6 FGDs with 58 participants. Data were collected by the first author, and six research assistants. All data collectors were bilingual (English and Hausa speaking).

### Data resource

**Data collection tool:** a 5-minute researcher-designed questionnaire was used to collect sociodemographic information (age, education, obstetric history), and an FGD guide with semi-structured questions was used to facilitate data collection during FDGs. Example of semi-structured questions asked: Q1; first, tell us a bit about how you travel to the health facilities. Probes: are the facilities too far from where you live or hard to get to? Do you have to pay for transportation? Q2: how did you feel when you came to the facility to deliver your baby? If you have never delivered in the facility, tell us about the experience of others. Probes: what usually happens? Are you seen right away, or do you have to wait long? Why is that the case? What do you think we should do to improve this? Do you feel that the staff treated you with respect and valued your point of view?

**Data collection:** FGDs were conducted either in outdoor settings or private rooms within the premises of a selected primary healthcare facility during non-working hours. Only researchers and participants were present during the discussions. All FGDs were conducted in Hausa (the participant-preferred and predominant language of the region), with sessions being audio recorded, and notes taken after obtaining with the consent from participants. No repeat FGDs were conducted, and participants were not required to give feedback on findings. Duration of FGDs ranged from 40-90 minutes, and participants were given refreshments worth 500 Nigerian Naira (NGN), equivalent to USD1.3, at the end of each FGD. Daily debriefing sessions were held with data collectors (first author, and six research assistants) to discuss findings and identify saturation of themes. Data saturation was reached within the six FGDs. The six research assistants who conducted and observed the FGDs translated recordings from Hausa to English and manually transcribed the data in English. For quality control, after the transcription of the first 2 FGDs, the first author (MAM) reviewed the transcripts against the respective audio recordings to verify the quality of the translation and transcription. The transcription process continued as transcriptions were considered satisfactory.

**Figure 1:**
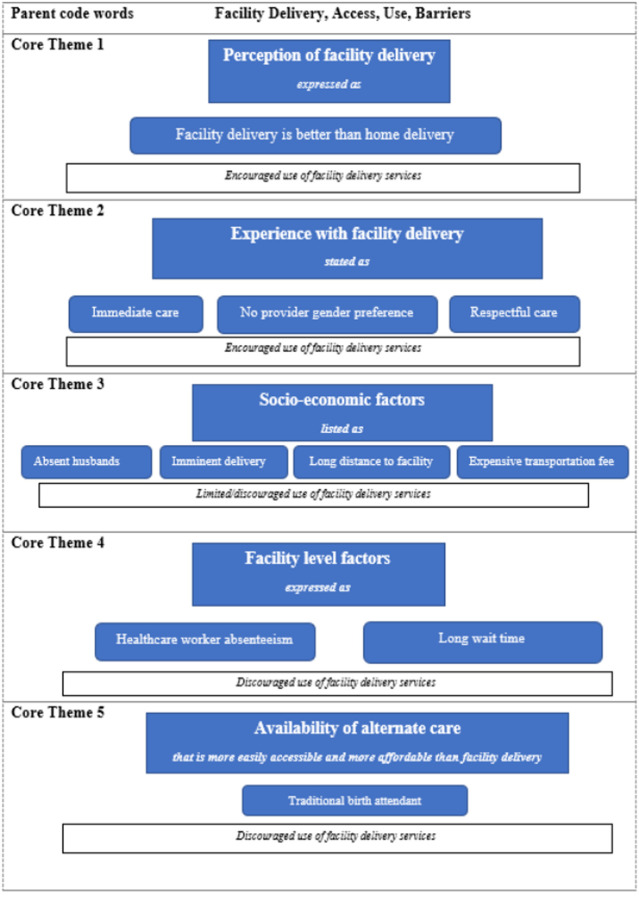
core themes in women's experiences with facility delivery services

**Data management and analysis:** all six transcripts were organized in NVivo 12 (Pro for Windows) and analyzed using a directed approach to content analysis. This analysis method allows the use of the literature to guide the initial coding scheme and/or relationships between core themes [29]. Therefore, keywords from the FGD guide, designed in line with previous findings from similar research, guided the initial coding scheme. Core themes used for coding: “experience with facility delivery services”, “facilitators to accessing facility delivery services”, “facility close to home”, “friendly healthcare workers”, “good care at facility”, “incentives to facility delivery”, “facility delivery is better than home delivery”, “getting immediate care at facility”, “good delivery care at facility”, “prefer female health workers”, “prefer male health workers”, “okay with male or female health workers”, “barriers to accessing facility delivery services” (Figure 1). All transcripts were analyzed through a deductive approach until no new theme emerged from the data. Thereafter, a second coder (Olukolade Shobo, the second author), analyzed about 30% of the transcripts. There was an 85% inter-rater agreement between the two coders (MAM and SO). The codes were then grouped under the main emerging themes (Figure 1).

**Ethical considerations:** ethical approval for this study was obtained from the ethics committee of the Gombe State Ministry of Health. Participants who could write provided written consent, and those who could not write provided verbal consent.

## Results

**Sociodemographic, obstetric history, and place of last delivery:** qualitative data were collected from 58 women. Participants´ mean age was 25.1 (± 5.3) years old. Banganje North participants were older than those from Akko and Zange Wards. Over half (59%) of the participants had been exposed to secular education. Approximately half of the participants (51%) were unemployed. The majority religion was Islam (69%), and the Fulani were the most prevalent ethnic group (36.2%). All participants (100%) were married, 76% had 1-4 children, and 64% delivered at a health facility within the last 12 months (Table 1).

**Table 1:** sociodemographic, obstetric history, and place of last delivery

Sample Size	N=7	N=11	N=10	N=10	N=10	N=10	N=58
Age, years: mean (SD)	28.0 (± 4.0)	30.4 (± 4.2)	24.0 (± 3.0)	25.0 (± 5.0)	21.1 (±4.4)	23.0 (± 4.1)	25.1 (± 5.3)
**Other characteristics: n (%)**							
Village healthcare worker intervention ward	Baganje North focus group 1	Baganje North focus group 2	Akko facility group	Akko home group	Zange facility group	Zange home group	All selected wards
**Place of last delivery**							
Facility	7 (100.0)	11 (100.0)	9 (0.0)	0 (0.0)	10 (100.0)	0 (0.0)	37 (63.7)
Home	0 (0.0)	0 (0.0)	1 (10.0)	10 (100.0)	0 (0.0)	10 (100.0)	21 (36.2)
**Formal education**							
None	1 (14.3)	1 (9.0)	1 (10.0)	3 (30.0)	4 (40.0)	0 (0.0)	10 (17.2)
Informal schooling^a^	0 (0.0)	0 (0.0)	5 (50.0)	0 (0.0)	0 (0.0)	9 (90.0)	14 (24.1)
Primary school	0 (0.0)	3 (27.0)	2 (20.0)	4 (40.0)	4 (40.0)	1 (10.0)	14 (24.1)
Secondary school	6 (85.0)	7 (64.0)	2 (20.0)	3 (30.0)	2 (20.0)	0 (0.0)	20 (34.4)
**Marital status**							
Single	0 (0.0)	0 (0.0)	0 (0.0)	0 (0.0)	0 (0.0)	0 (0.0)	0 (0.0)
Married	7 (100.0)	11 (100.0)	10 (100.0)	10 (100.0)	10 (100.0)	10 (100.0)	58 (100.0)
Polygamous union^b^	0 (0.0)	0 (0.0)	6 (60.0)	3 (30.0)	5 (50.0)	5 (50.0)	19 (32.7)
**Employment status**							
None	1 (14.3)	5 (45.0)	3 (30.0)	8 (80.0)	9 (90.0)	4 (40. 0)	30 (51.7)
Business/trade	1 (14.3)	0 (0.0)	7 (70.0)	2 (20.0)	0 (0.0)	6 (60.0)	16 (27.5)
Professional^c^	0 (0.0)	0 (0.0)	0 (0.0)	0 (0.0)	1 (10.0)	0 (0.0)	1 (1.7)
Farmer	5 (71.0)	6 (55.0)	0 (0.0)	0 (0.0)	0 (0.0)	0 (0.0)	11 (18.9)
**Religious affiliation**							
Christianity	7 (100.0)	11 (100.0)	0 (0.0)	0 (0.0)	0 (0.0)	0 (0.0)	18 (31.0)
Islam	0 (0.0)	0 (0.0)	10 (100.0)	10 (100.0)	10 (100.0)	10 (100.0)	40 (69.0)
**Ethnicity**							
Fulani	0 (0.0)	0 (0.0)	8 (80.0)	9 (90.0)	2 (20.0)	2 (20.0)	21(36.2)
Tangale	7 (100.0)	11 (100.0)	0 (0.0)	0 (0.0)	0 (0.0)	0 (0.0)	18 (31.0)
Others^d^	0 (0.0)	0 (0.0)	2 (20.0)	1 (10.0)	8 (80.0)	8 (80.0)	19 (32.7)
**Number of living children**							
None	0 (0.0)	0 (0.0)	0 (0.0)	0 (0.0)	0 (0.0)	0 (0.0)	0 (0.0)
1-2	2 (28.0)	3 (27.0)	2 (20.0)	5 (50.0)	5 (50.0)	4 (40.0)	21 (36.2)
3-4	4 (57.0)	4 (36.0)	5 (50.0)	3 (30.0)	4 (40.0)	3 (30.0)	23 (39.6)
5+	1 (14.3)	4 (36.0)	3 (40.0)	2 (20.0)	1 (10.3)	3 (30.0)	14 (24.1)
**History of facility delivery**							
None	0 (0.0)	0 (0.0)	0 (0.0)	2 (20.0)	0 (0.0)	9 (0.0)	11 (18.9)
1-2	5 (71.0)	2 (18.0)	2 (20.0)	6 (60.0)	7 (70.0)	1 (10.0)	23 (39.6)
3-4	2 (28.5)	5 (45.0)	5 (50.0)	1 (10.0)	3 (0.0)	0 (0.0)	16 (27.5)
5+	0 (0.0)	4 (36.0)	3 (30.0)	1 (10.0)	0 (0.0)	0 (0.0)	8 (13.7)
**History of home delivery**							
None	3 (43.0)	11 (100.0)	7 (70.0)	0 (0.0)	6 (60.0)	0 (0.0)	27 (46.5)
1,2	0 (0.0)	0 (0.0)	3 (30.0)	8 (80.0)	2 (20.0)	3 (30.0)	16 (27.5)
3+	4 (57.0)	0 (0.0)	0 (0.0)	2 (20.0)	2 (20.0)	7 (70.0)	15 (25.8)
**Use of a traditional birth attendant**							
None	4 (57.0)	11 (100.0)	9 (90.0)	2 (20.0)	6 (60.0)	0 (0.0)	32 (55.1)
1,2	3 (43.0)	0 (0.0)	1 (10.0)	7 (70.0)	2 (20.0)	3 (30.0)	16 (27.5)
3+	0 (0.0)	0 (0.0)	0 (0.0)	1 (10.0)	2 (20.0)	7 (70.0)	10 (17.2)
**Last delivery**							
Less than a month ago	0 (0.0)	1 (9.0)	2 (10.0)	2 (20.0)	0 (0.0)	0 (0.0)	5 (8.6)
1-3 months ago	3 (43.0)	3 (27.0)	2 (20.0)	6 (60.0)	3 (30.0)	1 (10.0)	18 (31.0)
4-6 months ago	2 (28.5)	1 (9.0)	2 (20.0)	1 (10.0)	4 (40.0)	5 (50.0)	15 (25.8)
7-9 months ago	0 (0.0)	3 (27.0)	3 (30.0)	1 (10.0)	2 (20.0)	2 (20.0)	11 (18.9)
10+ months ago	2 (28.5)	3 (27.0)	1 (10.0)	0 (0.0)	1 (10.0)	2 (20.0)	9 (15.5)
No response	0 (0.0)	0 (0.0)	1 (10.0)	0 (0.0)	0 (0.0)	0 (0.0)	1 (1.7)
**First contact with VHW**							
Less than a month ago	0 (0.0)	0 (0.0)	0 (0.0)	0 (0.0)	0 (0.0)	0 (0.0)	0 (0.0)
1-3 months ago	1 (14.2)	1 (9.0)	0 (0.0)	0 (0.0)	0 (0.0)	0 (0.0)	2 (3.4)
4-6 months ago	1 (14.2)	0 (0.0)	2 (20.0)	3 (30.0)	0 (0.0)	0 (0.0)	6 (10.3)
7-9 months ago	2 (28.5)	1 (9.0)	2 (20.0)	4 (40.0)	1 (10.0)	0 (0.0)	10 (17.2)
10+ months ago	3 (43.0)	9 (82.0)	5 (50.0)	3 (30.0)	9 (90.0)	10(100.0)	39 (67.2)
No response	0 (0.0)	0 (0.0)	1 (10.0)	0 (0.0)	0 (0.0)	0 (0.0)	1 (1.7)

SD: standard deviation; ^a^: Islamic or Bible School; ^b^: calculated as a subset of married women: 19 married women with either one, two or three co-wives; ^c^: one traditional birth attendant; ^d^: include four Boboriya, three Hausa, three Karekare, three Bolewa, two Kanuri one Waja and one Tera; VHW: village health workers

**Focus group discussion findings:** the five core themes that emerged from data analyses included: 1) Perception of facility delivery services; 2) experience with facility delivery services; 3) socioeconomic factors that limited/discouraged the use of facility delivery services; 4) facility-level factors that discouraged the use of delivery services; and 5) availability of alternate care (Figure 1).

**Perception of facility delivery services:** facility delivery better than home delivery - most study participants believed the care the mother-infant pair received at the facility during delivery was better than the care accessible to them during home delivery. They acknowledged that in the facility, there is medication available to prevent or arrest obstetric complications and to protect the health of the mother-infant pair. They were aware that these specialized drugs were not usually available to women who deliver at home. Participants also recognized that when obstetric complications arise because of home delivery, the mother-infant pair is eventually taken to the facility for specialized care. This report is supported by participant exemplar quotes below: “*There is a difference between facility care and care at home. At the facility, they use drugs that stop bleeding, help in resolving retained placenta issues, and drugs to dry the naval of the newborn. They clean up the child immediately after delivery, but this is not the practice at home*”, 23-year-old, Akko home group. ”*At the facility, the baby and mother are well taken care of, but at home, if any emergency arises, they will still need to be brought back to the facility for proper care*”, 18-year-old, Zange home group. Some participants also acknowledged that home deliveries could lead to maternal and neonatal mortality: “... *When you deliver to home and start to bleed, you could die at home, but if you deliver at the facility, the health workers will know how to treat you..*.” a 33-year-old, Banganje North facility group 2. “*The difference is that sometimes the child delivered is covered with leather* (amniotic sac) *like a ball. If it is in the facility, the sac will be slit immediately, to enable the child to breathe but if it is at home, it will be difficult for them to realize, and the infant may eventually die…*” a 22-year-old, Banganje North facility group 1.

**Experience with facility delivery:** participants indicated that they received timely, respectful maternal care in facility delivery services. Although most participants preferred to be cared for by female healthcare workers, they would not refuse the services of male healthcare workers.

Immediate care - some participants expressed that they received immediate care at the facility when they came to deliver without having to wait long hours. Even in instances when the healthcare workers were away from the facility, they rushed to get to the facility when a woman in labor required their service. As stated by one participant: “*They take good care of me. Immediately I arrived, I was received, and they begin their examinations. They do everything for you until you deliver safely*” 31-year-old, Akko home group. “*Once you come for delivery and the healthcare workers are informed, they will even come running to attend to you. Even if they are in their homes and they hear a car stop, and they are informed, they will even run to the facility*” 25-year-old, Akko facility group.

Some participants also expressed that healthcare workers were generally available in the facility even at night to attend to emergency cases, as stated by two participants: “*They [healthcare workers] are always around, even at night, to attend to emergencies*”, a 31-year-old, Akko facility group. “*When I started experiencing labor, I was conveyed to this hospital at about 3: 00 a.m. I met the nurse on night duty, and she took care of me*…” a 35-year-old, Banganje north group 1. Respectful maternal care - in all six focus groups, participants indicated that the healthcare workers at the facility treated them with dignity and respect, and they did not experience any negative treatment from them. “*I feel happy because they give me care and respect, they don´t have any problem*” a 24-year-old, Zange facility group. “*When I was brought to the facility when I was in labor, when I came in, the nurse who received me received me with a smile and laughter and asked me what the problem? I told her I was in labor. When I told her I was in labor, she placed me on the bed, she examined my abdomen, and when she examined my abdomen, she checked my BP* [blood pressure] *and kept me on the bed to rest*”, a 30-year-old, Banganje north group 1.

Most of the participants were satisfied with the quality of services they received from the healthcare workers at the health facility during delivery. “*I was taken to the health facility when I started labor, the health worker who received me was with me throughout the process and kept checking on me at regular intervals till I delivered safely. She took very good care of me till I delivered, and I delivered successfully*”, a 30-year-old, Banganje north group 2. “*No bad treatment, they* (facility healthcare workers) *treat us well and give us all the assistance that we seek*" all participants Banganje north group 1.

Healthcare worker gender preference - most participants within the six focus groups preferred female healthcare workers to assist them during delivery. Their preference was not associated with any cultural or religious requirements, but with the comfort and familiarity associated with being consulted by same-gendered healthcare workers. As one participant stated: “*I would prefer a woman, not because of religion or culture, but because a woman is my sister I can tell her anything but if it´s a man, I will be shy to talk to him…*” a 24-year-old, Zange facility group.

Some participants also believed that because female healthcare workers have experienced pregnancy, labor, and delivery, they would be more understanding and compassionate towards them during the labor and delivery process compared to male healthcare workers: “*I will prefer a female health worker to attend to me because I will have this belief, she knows what I´m going through and knows how to relate with me better than a male health worker who has not experienced labor before*”, a 23-year-old, Akko home group. However, some participants expressed that though they preferred female healthcare workers to assist them during delivery, they would not refuse to be assisted by a male healthcare worker. For other participants, their satisfactory experiences with both female and male healthcare providers led them to not be inclined to the gender of the health worker, but rather the quality of service. These views were expressed in the following participant quotes: “*I like both males and females because they have attended to me during my delivery. When I gave birth to my first daughter…, it was a male who attended to me, but now it is a female who attended to me*. [I prefer] *both*”, a 25-year-old, Banganje north group 1. “*For me, anyone* [male or female] *on duty can take my delivery*”, a 25-year-old, Zange home group. “*Anybody* [male or female] *can take my delivery as far as God gives me good health*”, a 20-year-old, Zange facility group.

**Socio-economic factors that limited/discouraged the use of facility delivery services:** barriers to accessing facility delivery services for some women included distance from the facility, expensive transportation costs, imminent delivery, and unavailability of husbands to accompany them to the health facility at the onset of labor. Geographical/financial constraint - some participants stated that even when they were aware of the health benefits associated with the use of facility-based MNH services and wanted to use the services, expensive transportation and facility user fees could limit their access and use of the services. As some participants eloquently expressed: “…*money can be a problem for those who are far because they need to pay for transportation and other necessities when they come to the hospital, like drugs and other things, so at times if they remember this they feel discouraged to come to the health facility*” a 24-year-old, Zange facility group. “…*some women are willing to go to the hospital, but a lack of money is what is stopping them*”, a 30-year-old, Zange facility group. However, when facilities were located proximal to a woman´s residence, they walked to the facility to deliver. As one participant stated: “*If the facility is close to us, everyone will have easy access to it and will deliver in it*”, a 23-year-old, 2 FDs and 0 HD, Banganje north group 2.

Easy access to transportation (family-owned, emergency transport scheme, or commercial vehicle/motorcycle) to travel to the facility facilitated the use of facility delivery services. However, the use of an emergency transport scheme, which is cost-free, was cited as more convenient in comparison to commercial transportation services that are associated with a cost. “*I use a bike to come because it is a bit far, so I do pay for transport*”, a 15-year-old, Zange facility group. “*When I started feeling the labour, we called the ETS and I was conveyed to the facility*”, a 22-year-old, Banganje north group 1. “*Emergency transport scheme is free, we don't pay*”, a 23-year-old, Banganje north group 2.

Imminent delivery - even when women and their family members (husbands and mothers-in-law) were aware and appreciated the value of using facility delivery services, some women inevitably delivered at home when the delivery was imminent. For some women, before the VHW arrived in their homes to accompany them to the facility, they would have already delivered. For others, the delivery occurred before they were transported to the facility. Participants with a history of home deliveries iterated the reasons they delivered at home in the quotes below: “*Delivery at a facility and ANC at a facility is better than home delivery. …before I could get a car to access facility services I delivered at home, if not I would not have delivered at home..*.”, a 31-year-old, Akko home group. “*She* [my mother-in-law] *likes it, but when am in labor and they call her* [VHW] *before she gets to my place, I already deliver my baby*”, a 20-year-old, Zange home group. However, some participants stated that when they inadvertently delivered at home and ended up with an obstetric complication such as a retained placenta, they accessed the facility so that the healthcare workers could assist in expelling the placenta. As some participants stated: “*Yes* [I delivered at home] *when you call them* [emergency transport scheme drivers], *they respond even if one deliver at home where one have delay placenta, you can utilize this drivers to convey you to the facility to have this placenta removed*”, a 31-year-old, Akko home group. “…*before I could get a car to access the facility services I delivered at home, if not, I would not have delivered at home. When I delivered at home, I came to the facility because of retained placenta*”, a 31-year-old, Akko home group.

Non-availability of husbands - when husbands were unavailable to accompany the wife to the facility at the onset of labor, the women ended up delivering at home. As some participants stated: “*He* [my husband] *is not always around* [to accompany me to the facility], *so whenever I am in labor I call on the TBA who live close to my house instead of going to the facility for delivery*”, a 20-year-old, Zange home group. “*I didn´t deliver at the facility because there was no one to bring me to the facility when I was in labour*”, a 25-year-old, Zange home group.

**Facility-level factors that discouraged the use of facility delivery services:** facility-level factors that limited the use of delivery services included healthcare worker absenteeism and long wait times.

Healthcare worker absenteeism - some women were unable to use the facility's delivery services when healthcare workers were unavailable in the facility. Healthcare workers were usually absent when they were on official leave or during statutory holidays. Consequently, these women ended up delivering before they could access another facility (usually located at a further distance than the facility they initially intended to use). Other women resorted to using the services of a TBA at home. As stated by some participants: “…*I delivered at home because of the health workers' strike, before I could get a car to access facility service elsewhere, I delivered at home*”, a 23-year-old, Akko home group. “*I had my two kids during Christmas when all the staff were on break*” a Zange home group.

Long waiting time at the facility - some participants indicated that when healthcare workers do not attend to them in a timely manner during ANC appointments, the delay leads to long waiting times that could drag into late-night hours. When this occurs, and women stay out late, their husbands become reluctant to allow them to access facility services in the future. As one woman explained: “…*I want them to improve on ANC, because when women come they don´t attend to us until the women become plenty and they will find it hard to attend to us on time, some women end up going home late at night and husbands won´t allow their wives go to the hospital again*”, a 22-year-old, Zange facility group.

**Availability of alternate care discouraged use of facility delivery services:** traditional birth attendants (TBAs) - in Zange Ward, where the TBAs were still actively functioning as local healthcare service providers, some women used facility services for antenatal care (ANC) but delivered at home with the assistance of the TBA. One participant´s statement affirms this: “*We always come for antenatal, but when it´s time for delivery we have our TBA who attends to us at home*”, a 20-year-old, Zange home group.

## Discussion

Our study indicated that most women had positive experiences with, and preferred facility birth to home birth. Participants and their significant family members (husbands and mothers-in-law) generally preferred and believed facility delivery was better than home delivery in terms of care and health benefits available for the mother-infant pair. Though most participants preferred a female healthcare worker to assist them during delivery, they would not refuse the services of a male healthcare worker. Factors that limited/discouraged facility delivery included long distances to care, financial vulnerability, imminent delivery, non-availability of husbands at the onset of labour, long wait times at the facility, healthcare workers´ absenteeism, and availability of alternate care (TBAs).

Facility delivery was considered more beneficial for the health of the mother-infant pair in comparison to home delivery. Similar findings were reported in an earlier study with women of reproductive age in North-Central Nigeria [30]. Furthermore, most of our participants expressed satisfaction with the timely, respectful reception and quality of services they received at the facility. Nonetheless, some participants from Zange Ward suggested that healthcare workers should have a more positive attitude towards women. This suggestion aligns with earlier reports that indicated healthcare worker unfriendliness and mistreatment were major factors that discouraged women´s use of facility delivery services in low-resource settings, including in Gombe State [20,31]. Furthermore, our participants´ preference for a competent healthcare worker, regardless of the healthcare workers´ gender, was also reflected in another study in North-Central Nigeria [30]. These findings could imply that aligning patients with the same gender healthcare worker might not be necessary to improve facility delivery uptake.

Some women attended ANC appointments in facilities, but deliberately resorted to TBA services for delivery. There might be three possible reasons for this pattern of behaviour. The first reason could be that TBAs were more friendly and respectful of women´s cultural values when providing care, unlike the healthcare workers in the facility [18,32]. The second reason women might prefer TBAs to healthcare workers for delivery could be that women were unsatisfied with the quality of care they received at the facility during delivery [33]. This dissatisfaction could be associated with healthcare worker attitude, understaffed facilities, inadequate infrastructure, and/or essential equipment/commodities [31,34]. The third reason women might prefer TBAs to healthcare workers for delivery could be that TBAs are easily accessible within the community. This proximity could imply that women did not have to travel a long distance or pay a transportation fee to access a TBA´s services. Therefore, in order for facility services to compete more favorably with TBA services, the MNH project should consider training TBAs to help in creating demand for facility delivery services through advocacy [35]. To further encourage the use of facility delivery services, facility health committees (constituting community members), which appear to have a positive influence on the quality of maternal and child health services in Northern Nigeria, could be integrated into the MNH project [35]. The committee could be charged with finding innovative solutions to problems encountered by women in health facilities, as well as educating the community on the value of facility maternal services on the health of the mother-infant pair [35].

Financial vulnerability was reported by our participants and other women in SSA [31,36] as a factor that limited the use of facility delivery services. This finding is not inapt, considering most of our participants (60% not engaged in an income-generating activity) from Akko and Zange seemed to be at a low socio-economic status. As a consequence, they would most likely find it difficult to pay an out-of-pocket fee to use facility delivery services [4]. Eliminating a facility user fee would most probably increase the uptake of facility delivery services [13]. This relationship between free healthcare service and optimum use of facility delivery services could be demonstrated in Banganje North Ward. Participants from that ward stated that facility delivery was cost-free, and that the ward had the highest uptake of facility delivery services.

Healthcare worker absenteeism was reported by our participants and other women in SSA [31,33,36] as a factor that discouraged the use of facility delivery services. Healthcare worker absenteeism in facilities could imply that some facilities were either grossly understaffed or were not functioning 24 hours a day [18,32]. Considering that healthcare worker absenteeism was not mentioned as a factor that discouraged the use of facility delivery services among participants from Banganje North Ward could imply that facilities in Banganje North probably functioned 24-hours a day and/or managed their human resources in such a way that there was always a healthcare worker available to attend to a woman in labor. As reported by Hussein *et al*. (2016), understaffed and/or non-24-hour functioning facilities usually discouraged the use of facility delivery services [33]. This discouragement is rooted in the fact that women would not like to travel to a facility to either meet no one to attend to them, or have to endure long wait times [33]. Long clinic wait times were also cited as a factor that discouraged use of facility delivery services for some participants in our study, as well as in other countries in SSA [31]. Considering that long clinic wait times could be related to personnel understaffing [18,31]. It is vital for MNH facilities to be adequately staffed, and clinic wait times should be lively and engaging through singing and dancing, to alleviate women´s dishearteningly long waits at the facility.

Imminent birth was reported as a factor that limited the use of facility delivery services among participants from Akko and Zange Wards. The prevalence of imminent birth could be related to the fact that some facilities were far from the women´s residence. Therefore, the incremental time required to arrange for transportation to the facility after the onset of labor could prolong the facility's arrival time. Another factor that could be responsible for imminent delivery is socio-economic vulnerability. The role of socio-economic vulnerability and the use of facility delivery services was illustrated among our study participants. For instance, the socio-economic status of participants from Akko and Zange Wards (secular education: 45%, occupation: 37.5%), with average (65%) and low (23%) uptake of facility delivery services respectively, are lower than those of Banganje North participants (secular education: 89% occupation: 67%) with the highest uptake (96%) of facility delivery services. Furthermore, participants from Banganje North (29 years old) were older than participants from Akko and Zange (23 years old) (Table 1). This finding aligns with the well-known fact that more educated and socio-economically empowered women have greater odds of using facility delivery services compared to younger, less socio-economically empowered women [37-40]. Therefore, we can infer that imminent birth as a factor that limits facility delivery is possibly related to women´s socio-economic vulnerability and lack of birth preparedness, and not necessarily an imminent physiological occurrence. Therefore, to prevent imminent births that usually occur at home, village health workers should be encouraged to educate women and their families (especially husbands) on the dangers of home deliveries and the possible adverse effects of delivering at home with the assistance of a TBA on the health of the mother-infant pair.

Unavailability of husbands at the onset of labor was stated as a factor that limited the use of facility delivery services. This form of women´s social dependency on their husbands could be associated with the patriarchal nature of most communities in Gombe State [41]. In most of the states, women require their husbands´ permission or must be accompanied by him before travelling out of the home. This requirement is in line with cultural/religious laws common among Muslims [30,31,42]. The relationship between such cultural/religious laws and the use of facility delivery services could be demonstrated among the cultural/religious similarities and differences among our participants. For instance, participants from Akko and Zange Wards were mostly Muslim, while participants from Banganje North were predominantly Christian. This religious difference could be among the factors responsible for the lower uptake of facility delivery services in the two former groups in comparison to the latter group. Furthermore, this finding aligns with national data, which shows that uptake of facility delivery services is usually higher among Christians compared to Muslims [43,44]. Therefore, village health workers should especially target and advise Muslim men to delegate a family member/friend or a village health worker to accompany their wives to the facility when the need might arise in their absence. This recommendation is entrenched in the fact that evidence has shown that male partners´ approval/support is usually vital to the use of facility delivery services for the women [37,45].

**Study limitations and strengths:** typical of qualitative studies, our findings are not generalizable to the whole of Gombe State. However, considering many Gombe residents live in the same rural areas as our participants and face the same challenges with access and use of maternal health services, our findings can be generalized to populations with similar socio-demographic characteristics within the state. One main strength of our study is demonstrated in the fact that our findings were in line with the earlier collected quantitative data on facility delivery uptake among MNH intervention wards. In our findings, factors that limited/discouraged facility delivery use were mentioned by more participants in the ward with the least facility delivery uptake (Zange 23%), by fewer participants in the Ward with the average facility delivery uptake (Akko 65%) and mentioned by very few participants in the ward with the highest facility delivery uptake (Banganje North 96%). Furthermore, the fact that we could not recruit a home delivery group in Banganje North Ward further validates the quantitative data on facility delivery uptake. In the future, MNH project designs should be informed by the formative research findings from the populations they wish to serve.

## Conclusion

Our findings indicate that even within one state in Northeast Nigeria, the use of facility delivery services within an MNH program varied. This variation could be related to the differences in facility efficiencies, socio-economic, cultural/religious context women find themselves. Therefore, to optimize the value of the MNH project intervention, its implementation cannot be a top-down approach or a one-size-fits-all. Factors that limit/discourage the use of facility delivery services in every setting must be assessed, and strategies to overcome those challenges should be integrated into the MNH project in a culturally sensitive manner.

### What is known about this topic

- Nigeria's high maternal and neonatal mortality rates are partly due to sub-optimal use of facility delivery services;
- A maternal neonatal health (MNH) program was implemented in Gombe State to mitigate the barriers (healthcare worker insufficiency, transportation challenges, and unavailability of essential lifesaving drugs) to the use of facility delivery services;
- Even within the context of the MNH program, some women still encountered barriers to using facility delivery services.

### What this study adds

- Barriers to facility delivery include use of traditional birth attendants, absent husbands at the onset of labor, imminent delivery, long distance to the facility, expensive transportation fees, healthcare worker absenteeism, and long clinic wait times;
- MNH programs should endeavor to account for the unique socioeconomic and cultural uniqueness of the communities they are meant to serve; a one-size-fits-all might not be the most effective approach.
